# Efficiency of telephone triage in the assessment of low back pain at a tertiary spine clinic

**DOI:** 10.1186/s12891-025-09416-y

**Published:** 2025-12-17

**Authors:** Maria Rachevitz, Helen Razmjou, Susan Robarts, Albert Yee, Amy Wainwright, Patricia Dickson, Joel Finkelstein

**Affiliations:** 1 Bone and Joint Program, Sunnybrook Health Sciences Centre, Holland Orthopaedic and Arthritic Centre 43 Wellesley Street East, Toronto, ON M4Y 1H1 Canada; 2 Department of Physical therapy, Faculty of Medicine, University of Toronto, Toronto, Canada; 3 Sunnybrook Research Institute, Sunnybrook Health Sciences Centre, Toronto, Canada; 4 Robarts Healthcare Consulting Inc, Midhurst, Canada; 5 Department of Surgery, Division of Orthopedic Surgery, Sunnybrook Health Sciences Centre, Toronto, Canada; 6 Division of Orthopaedic Surgery, Department of Surgery, Faculty of Medicine, University of Toronto, Toronto, Canada; 7 Department of Occupational Therapy, Faculty of Medicine, University of Toronto, Toronto, Canada

**Keywords:** Virtual, Rapid access clinic, Spine, Telemedicine

## Abstract

**Purpose:**

In response to COVID-19 related health care access restrictions, telemedicine was introduced to continue to provide necessary health care access in Ontario, Canada. This study examined the role of a telephone assessment (triage) in streamlining referral consultation in patients with low back pain who were referred to a spine specialty surgical clinic.

**Methods:**

This was an observational study of patients presenting with low back pain with or without leg pain. The virtual interview was conducted by an experienced Advanced Practice Physiotherapist (APP) via telephone. The clinician documented the current symptoms, reviewed referral information including diagnostic imaging and made decisions regarding surgical appropriateness. Patients with cauda equina syndrome, metastatic spinal cord compression lesions, infection and fractures were excluded. Descriptive analysis and general linear modeling were conducted.

**Results:**

A consecutive sample of 100 patients, 50 females (average age = 58, SD: 16, range 20–87 years) participated in the virtual assessment. Of 100 participants, 41 (41%) were deemed appropriate to proceed for an in-person assessment, with 36% being considered as potential surgical candidates (33 patients were seen by a spine surgeon and 3 were referred to a hip surgeon for hip arthritis). Fifty-nine (59%) patients did not require an in-person visit to the clinic. The most prominent reason for subsequent surgical assessment was leg dominant pain (neurogenic claudication/radiculopathy) with concordant imaging findings (27, 75%). There was a statistically significant association between surgical candidacy and presence of leg symptoms secondary to disc herniation or claudication (*p* = 0.0002) with no association with the scores of the PROMs or isolated imaging (*P* > 0.05).

**Conclusion:**

A structured virtual telephone interview was an effective means of triaging patients with LBP with and without leg pain with a potential of reducing the number of unnecessary visits to a spine surgeon. Radicular pain to the lower extremity was the most common symptom among participants who were directed to the surgeon.

**Supplementary Information:**

The online version contains supplementary material available at 10.1186/s12891-025-09416-y.

## Introduction

Low back pain (LBP) is one of the most prevalent causes of musculoskeletal disability worldwide with a significant cost and burden on health care systems [1]. Most patients with non-specific LBP seek help from their primary care providers who often prescribe pain medications and physiotherapy. In Ontario, publicly funded physiotherapy services are limited and many patients may not have the means to pay privately. This in combination with historically long wait lists for surgical consultation (made worse by the COVID-19 related restrictions) may result in prolonged disability and a reliance on pain medication. To facilitate access, new practices were introduced in spine care to avoid prolonged disability and expedite the triage of consultations for patients who are referred to a surgical clinic for care. One of these new initiatives was the introduction of telemedicine to screen and triage patients referred to a spine clinic for surgical consult.

Telemedicine refers to a remote communication with an audio and/or visual device. With increased access to modern communication technology and restrictions following COVID-19 pandemic, telemedicine saw a notable rise in utilization in clinical practice [2–9]. Telephone is the most accessible mode of communication with patients who have limited access to video technology, or internet connectivity. A phone conversation by a skilled regulated health professional with advanced training, allows for assessment of patient’s symptoms and functional limitations. It facilitates discussion of treatment option while offering guidance and education for self-care. Research indicates telemedicine can reduce the cost of medical care without a negative impact on patient satisfaction [2–4]. The use of virtual care was initially implemented in response to the COVID-19 pandemic and was continued to provide a better means to expedite the management of patients referred with LBP in the post-pandemic era [5]. As it relates to non-traditional forms of clinical assessment, the limited publications indicate the popularity of virtual care among Canadians [6]. Providing preoperative instructions and notifications virtually has shown good adherence to those instructions in patients undergoing elective spine surgery [7] with good validity for spinal clinical examination components [8] and an overall high satisfaction reported for spine care [9]. Innovative modes of clinical care delivery need to be examined for safety and effectiveness and to this end, further assessment of a structured telephone triage in the assessment of LBP is warranted.

The primary purpose of this study was to examine the effectiveness of a structured telephone triage with the implementation of self-administered outcome measures on reducing the number of in-person spine clinic visits. The secondary purpose was to examine the relationship between surgical candidacy and self-report scores, performance outcome measures, and diagnostic imaging data collected via the phone interview.

## Methods

### Participants

This was an observational study of consecutive patients with LBP symptoms with or without leg pain, who had been referred to a specialty spine surgeon clinic for consideration of surgery in Ontario, Canada. Exclusion criteria included presence of red flags (cauda equina syndrome, metastatic spinal cord compression lesions, infection, fractures), or inability to communicate in English.

### Virtual clinical examination

A structured virtual telephone interview (Appendix A) was conducted by an Advanced Practice Physiotherapist (APP) with 22 years of clinical experience. Patients received instructions on the interview, a functional test, and self-reported surveys prior to the interview via email. The functional test was conducted by the patient at home. The APP had access to the completed survey results at the time of phone interview. The APP completed a pain diagram and obtained a thorough history of the mechanism of injury, onset, duration and type of symptoms, previous treatments, and patient perspectives on final management. The APP had access to plain radiographs, Computed Tomography (CT) scan, Magnetic Resonance Imaging (MRI) reports or images via a hospital-wide utilized software. The interview took about one hour.

### Patient Reported Outcome Measures (PROMs)

All patients were sent an email prior to the interview with 4 questionnaires: back vs. leg pain questionnaire [10]; 4 items from Self- administered self-reported spinal stenosis history (SSHQ) [11], two patient reported outcome measures (PROMs) questionnaires and instructions on how to perform and record the results of a functional test. Self -administered measures included the Oswestry disability Index (ODI) [12] and STarTBack [13]. The functional test was the 5-Repetition Sit-to-Stand test [14].

The ODI is a self-report low back functional outcome measure and includes 10 items addressing patient’s back or leg pain and ability to manage daily living. Each of the 10 items is scored from 0 to 5, with the maximum possible score being 50. The ODI severity was categorized as 0–20: no treatment, 21–40: moderate, > 40 severe disability, needs investigation and treatment. The ODI has demonstrated good psychometric properties in patients with spine conditions [12, 15–17].

STarTBack [13] is a validated screening tool designed to identify prognostic indicators relevant to clinician’s decision-making concerning initial treatment options in primary care. StarTBack has nine items, addressing both biomedical and psychosocial risk factors reflecting fear, anxiety, catastrophizing tendency, depression and bothersomeness. The total score ranges from 0 to 9 and is produced by summing all positive items. The psychosocial subscale score ranges from 0 to 5 and is produced by summing the answers of question 5 to 9. Patients with total score of < 3 are classified as low risk; A minimum of 4 points on total score and < 3 on the psychosocial subscale indicates a medium risk. A score of 4 or more on the total score and 3 or more on the psychosocial subscale is classified as high risk for poor prognosis and persistent disability. STarTBack has been validated for primary care and tertiary settings [18–21].

### 5-repetitions sit-stand functional test

This functional test was conducted by asking patients to sit down on a standard height chair with a straight back with their arms folded crossed across their chest and to keep their feet flat on the floor. Patients were instructed to fully stand and sit down without using their upper limb and time the test by a stopwatch from the beginning to the completed fifth stand. Patients who could not complete the test received a zero score [14, 22–24].

### Clinical symptoms and diagnostic categories

The questionnaires were used in combination with focused spine history, pain diagram and review of the imaging report. Pain pattern was documented as low back dominant or leg dominant. Leg pain was then subcategorized as claudication vs. radicular type leg pain. Patients provided information on easing and aggravating factors (e.g. rest vs. activity and bending vs. walking).

Claudication is defined as leg pain associated with walking, a common symptom reported by elderly [25]. Symptoms are described as burning, cramping, numbness, and weakness. Intermittent claudication can be neurogenic or vascular. Neurogenic claudication is caused by lumbar spinal stenosis (LSS) when a narrowed and degenerative stenotic spinal canal or foramen is further compromised by extended lumbar posture, leading to direct mechanical compression or indirect vascular compression of the nerve roots and/or cauda equina [26]. Radiculopathy was categorized as claudication type and discogenic. The neurogenic claudication is characterized as diffuse symptoms, often bilateral and is associated with central canal stenosis. The claudication-type radiculopathy has a dermatomal distribution, often involving one leg. The unilateral radicular symptoms secondary to spinal stenosis are often related to stenosis of the lateral recess or the foraminal canal [27].

Clinical diagnosis made by the APP was based on combined information on pain pattern, aggravating/easing factors, and imaging findings and was documented in four categories: (1) degenerative disc disease/mechanical low back pain, (2) radiculopathy (claudication type and/or discogenic), (3) neurogenic claudication, and (4) other (e.g. hip/knee osteoarthritis).

### Radiological examination

The results of imaging were based on plain radiographs and advanced imaging (CT/MRI) and were categorized as central spinal canal stenosis, foraminal stenosis, lateral recess stenosis, disc herniation/bulge, facet joint cyst, spondylolisthesis, and other.

### Management plan

Management plan was based on the results of the PROMs, functional test, patients’ perspective, clinical and radiological findings. The APP discussed the results with the patient and explained their options including conservative and surgical management. Patients’ perspective was incorporated into the final decision and their choice of wanting to proceed with surgery was documented in the data collection form (Appendix A).

### Follow-up assessment

Patients were either discharged after the first interview, had an in-person visit with the APP or spine/lower extremity surgeon, or had a telephone follow-up based on the complexity of the condition.

### Sample size justification and statistical analysis

Previous research has shown that 77% of patients seen by an APP do not require surgical consultation [28]. Considering this was a virtual telephone interview and to increase precision, we hypothesized that reduction of the number of in-person visits by at least 20% would establish the effectiveness of the telephone triaging. To detect a difference of this size, we required 95 patients [29]. Descriptive statistics were calculated for demographic variables, self-report, performance score, and diagnostic imaging findings. The percentage of patients who did not see a surgeon was calculated. The relationship between dependent variable (surgical candidacy) and independent factors (demographics, ODI, 5-repetitation sit stand test, clinical and radiological findings) was explored using separate univariable general linear models (GLM) and F-tests. PROC GLM in SAS has many applications including analysis of variance for categorical data and simple linear regression for continuous data. The F-value of the F-test generates 1-tailed p values that indicate directionality in the hypothesis testing. Statistical analysis was performed using SAS^®^ version 9.4 (SAS^®^ Institute, Cary, NC).

## Results

A consecutive sample of 100 patients, 50 females, average age (58, SD: 16, range 20–87 years) participated in the virtual assessment. Table 1 shows the demographics and results of the triage by the APP. Of 100 participants, 41 (41%) were deemed appropriate to proceed for an in-person assessment: 36 patients were sent for surgeon consultation (33 by a spine surgeon and 3 by a hip surgeon), 5 patients required an in-person assessment by the APP, and 27 needed a follow-up phone assessment. Fifty-nine (59%) patients did not require an in-person visit to the clinic. There were 5 (13%) cases with signs of spondylolisthesis with segmental instability, and 3 (8%) cases of end stage hip arthritis requiring hip replacement.

**Table 1:** Patient characteristics and type of visit (*N* = 100)

Variables	*N* (%), Mean (SD)
Sex • Male • Female	50 50
Age (Y, mean, SD, range)	58(20), 20–87
Type of assessments • In-person visit with surgeon • In-person visit with APP • One time phone call assessment • Follow-up phone call by APP	36 5 32 27
Subcategories of surgical candidates (*N* = 36) **£** • Neurological • Structural lesions • Patient considering surgery • Complex presentation • Other joints	27 5 20 7 3
Primary diagnosis of surgical candidates (*N* = 36) • DDD/Mechanical LBP • Radicular pain • Neurological claudication • Hip Joint severe OA	2 26 5 3
Primary diagnosis of patients seen in-person by APP (*N* = 5) • DDD/Mechanical LBP • Leg-dominant pain, not exhausted conservative treatment	2 3
Primary diagnosis of patients with 2nd virtual appointment (*N* = 27) • DDD/Mechanical LBP • Radicular pain • Neurological claudication • Other	2 21 3 1

*APP* Advanced Practice Physiotherapist, *DDD *Degenerative disc disease, *LBP* Low back pain, *OA* Osteoarthritis

£: categories overlap

Radicular pain with concordant imaging findings were the most common features of participants who were directed to the surgeon (27, 75% of surgical sample). There were 5 (13%) cases with spondylolisthesis with signs of segmental instability, and 3 (8%) cases with end stage hip arthritis requiring hip replacement. Of the 33 patients who were sent to the spine surgeon, seventeen patients (17/33: 52%) ultimately had surgery (fusions, laminectomies with or without discectomy). Four (12%) were considered non-surgical. Five (15%) were referred by the surgeon for further investigations or treatment: one was diagnosed with ALS, one was referred for spinal stimulator, and three improved while waiting for surgical consult. Seven (21%) were not considered to be immediate candidates for surgery as they had not exhausted conservative treatment or were not ready to proceed with surgery due to medical or personal reasons at the time of the assessment.

**Fig. 1:**
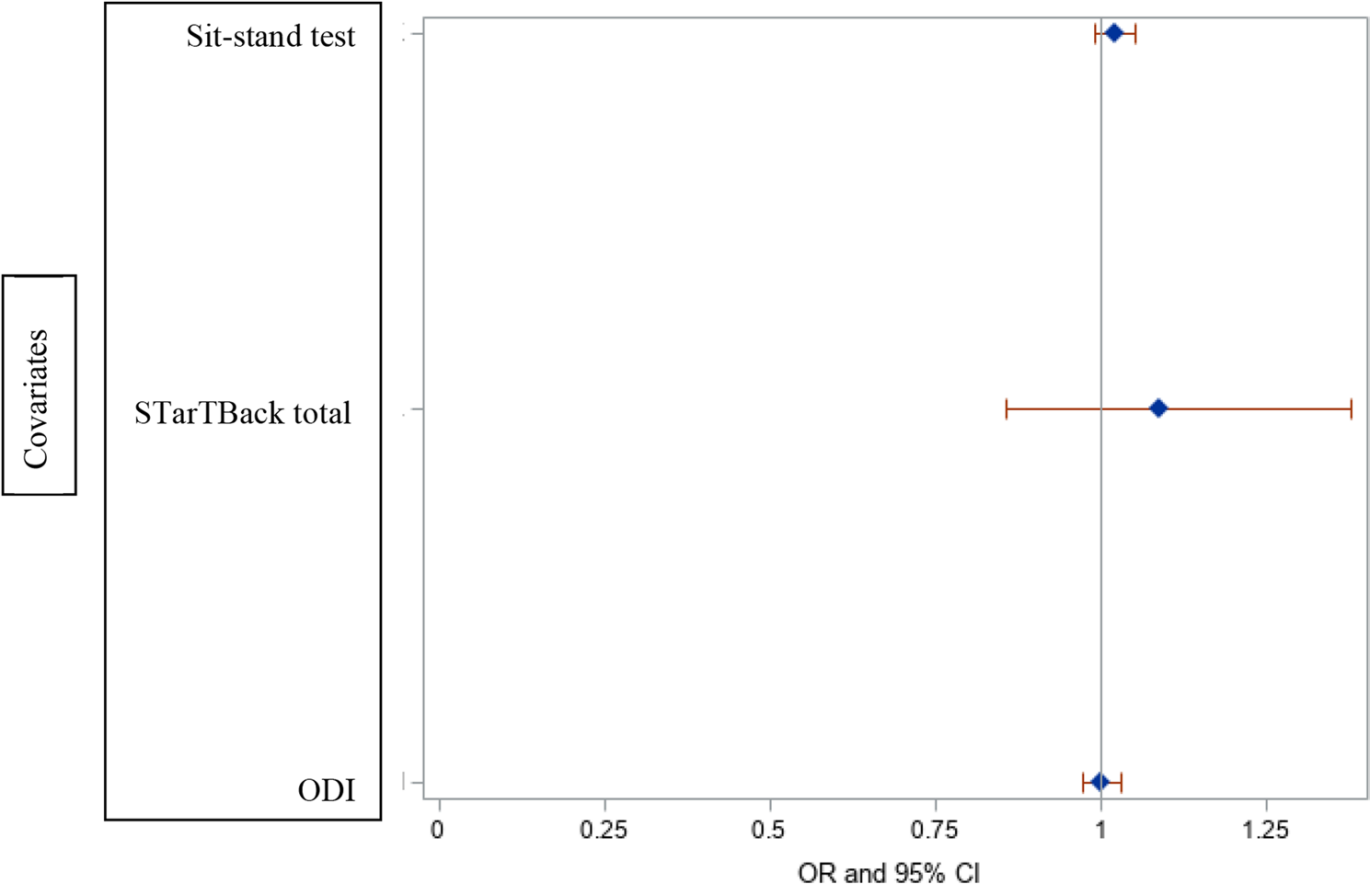
Forest plot of surgical candidacy and functional test, STarTBack and ODI scores

**Fig. 2:**
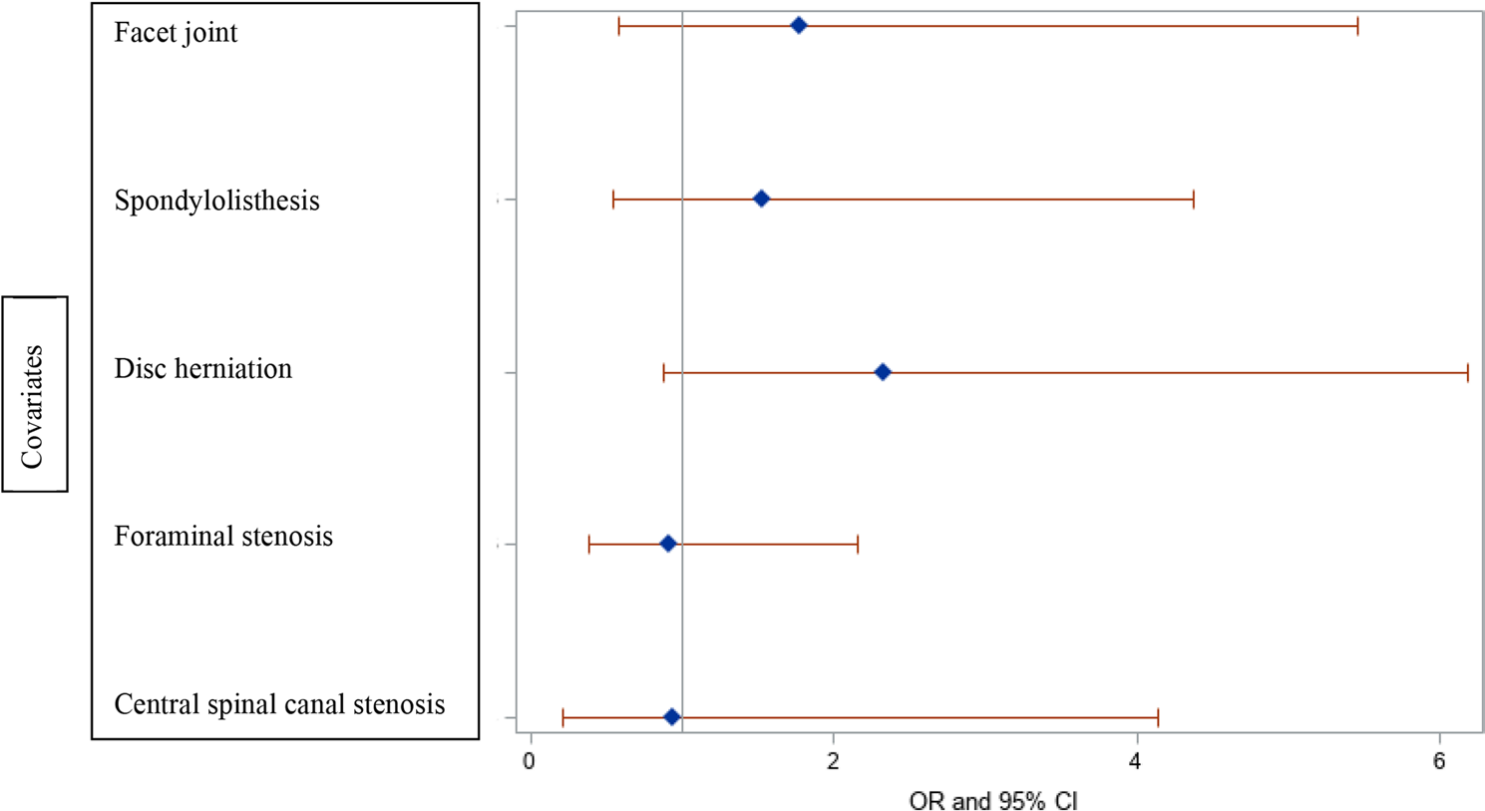
Forest plot of surgical candidacy and isolated imaging results

Table 2 shows the pain pattern categories, average of PROMs and imaging findings. While there was a significant association between surgical candidacy and leg symptoms secondary to disc herniation or claudication (*p* = 0.0002), there was no association between surgical candidacy and PROMs, the functional test, or the isolated imaging findings (Figures 1 and 2) (*P* > 0.05).

**Table 2:** Symptom pattern, proms and imaging findings (*N* = 100)

Variables (Mean, SD)			
Pain pattern • Back dominant • Leg dominant (claudication) • Leg dominant (nerve root) • Resolving • missing		44 (47%) 31 (33%) 18 (19%) 1(1%) 6	
PROMs • StarTBack total • Low risk • Medium risk • High Risk • ODI Total • No treatment • Moderate disability • Severe to bedridden		6.56 (SD = 2.37) 19 23 58 43.4 (SD = 19) 15 34 51	
Imagining Findings £			
• Formaninal stenosis 47(47%)		9(9%	
• Formaninal stenosis		47(47%)	
• Disc herniation		54(54%)	
• Spondylisthesis		21(21%)	
• Facet joint cysts		21(21%)	

STarTBack: Subgroups for Targeted Treatment Back screening Tool

STarTBack categories:

Low risk: <3, medium risk: 4 points on total score and < 3 on the psychosocial, high risk: ≥4 of total score and ≥ 3 of psychosocial subscale

*ODI* Oswestry Disability Index

ODI severity: 0–20: no treatment, 21–40: moderate, > 40 severe disability, needs investigation and treatment

£: categories overlap

## Discussion

Virtual care has become an essential part of the care pathway for patients with musculoskeletal conditions in the post COVID era [30–34]. The present study indicates that important components of the spine surgical triage can be obtained through a structured telephone interview, including patient’s pain pattern, symptoms, self-reported disability, and imaging findings. In the sample studied, three patients reported groin and leg symptoms related to advanced osteoarthritis of the hip joint that required total hip arthroplasty. Of all patients with a wide variety of symptoms and imaging findings that participated in the study, only 41(41%) required an in-person assessment (5% by the APP, 33% spine surgical consultation and 3% hip/knee surgical consultation). Fifty nine (59%) of patients did not require in person visits, therefore virtual triage reduced the requirement for in-person visits by 59%.

In the present study, neither of the PROMs or isolated imaging findings were solely or independently related to surgical candidacy. The scores of the PROMs and functional difficulties in combination with pain pattern and radiological findings, however assisted the APP with clinical diagnosis and establishing a need for surgical consultation. The APP was able to synthesize all components of the gathered information to reach a conclusion through clinical intuitive, often influenced by the clinical setting (primary vs. tertiary), disease prevalence, clinician’s experience, patient’s reported PROMs and clinical presentation, investigation results and other unique factors. The details on role of pre-test probability of a disease and how it is affected by different components of examination is beyond the scope of this paper and can be found elsewhere [35, 36]. In line with our study, apart from selecting suitable patients and having access to appropriate imaging modalities, using dermatomal and pain distribution charts and other tools have been reported to improve the feasibility of the virtual care [37].

The information on telephone triage is sparse at present. The results of studies on virtual spine care however, indicate improved access and quality of care, significant cost savings, and high patient satisfaction with near equivalent preference to in-person appointments [30–34]. Our study showed that patients were efficiently and safely assessed via a telephone interview and were directed to an appropriate clinician based on structured information that included both patient and clinician’s perspectives. In a study that involved 33 patients, the videoconference triage was reported to be feasible and provided an acceptable form of care for patients who were interested and able to participate in this form of care [30]. A systematic review of virtual spine examination published in 2021 [32] that included three studies [88 patients] comparing virtual with in-person spine physical examination has shown acceptable reliability for portions of the virtual exam. Patient satisfaction surveys were conducted in 2 of the studies and showed generally high satisfaction (> 80% would recommend). In a recent survey study of 243 physiotherapists in Ontario [33], respondents indicated that access to technology that met standards of safety, confidentiality, and privacy was an important factor to facilitate virtual care. Factors that encouraged patients to use virtual care were convenience and ease of scheduling sessions, and comfort of remaining in one’s home. Barriers were noted as negative attitudes, poor suitability, and internet connectivity. In a survey study of 485 spine surgeons [34], 39% of participants reported that the main challenge in using virtual care was the limited ability to perform a physical examination, followed by concerns of medico-legal implications (19%). Surprisingly, only 9% of participants reported problems with technology. In the same study [34], patients had a positive response to virtual clinics, feeling satisfied that virtual clinics were at least at the same level as face-to-face clinics.

In summary of our study, a virtual telephone assessment of patients with LBP by a trained APP is a feasible and efficient method of triaging patients for surgical consultation provided that the assessment is based on a structured history taking, symptom analysis, clinical and radiological review, and patient perspectives. Our results support the role of virtual triage which may complement standard practices to enhance the timelier triage of referred patients. By redirecting non-surgical patients to an appropriate care earlier, unnecessary visits to a spine surgeon will be reduced and actual surgical patients will receive an expedited care. It also highlights the ability of virtual triage to diagnosis non-spinal pathologies (e.g. OA hip).

### Limitations

The present study has some limitations. Our results apply to patients with lumbar spine pain with or without leg pain, referred to a tertiary spine clinic and do not apply to patients with serious problems such as the cauda equina syndrome, metastatic spinal cord compression lesions, infection, or fractures. The results may not apply to all therapists due to extensive training of the APP in this study and further examination of the efficiency of telephone triaging needs to be examined in a variety of settings such as community clinics. Case-control designs will provide more solid conclusions on value of this mode of assessment in comparison to in-person visits.

## Conclusions

A structured virtual telephone interview was an effective means of triaging patients with LBP with and without leg pain with a potential of reducing the number of unnecessary visits to a spine surgeon. Radicular pain to the lower extremity was the most common symptom among participants who were directed to the surgeon.

## Supplementary Information

Supplementary Material 1

Supplementary Material 2

## Data Availability

Data are available from the corresponding author on reasonable request.
