# Supplementary material for: Efficiency of telephone triage in the assessment of low back pain at a tertiary spine clinic
Source: BMC Musculoskelet Disord. 2025 Dec 17;27:104. doi: 10.1186/s12891-025-09416-y (PMC12874845; doi:10.1186/s12891-025-09416-y)
Supplement: Supplementary file 1 — Supplementary Material 1 [file 12891_2025_9416_MOESM1_ESM.docx]

Age:_______ SEX: F / M Symptom duration: __________months

## **Mechanism of Injury:**

❑ Insidious ❑Repetitive lifting/bending ❑Fall ❑Traumatic (MVA) ❑WSIB ❑Other __________

**Back-versus leg-pain questionnaire**

Q1. Pain Rating Low Back _____ Right Leg_____ Left Leg ______

Q2. Treatment at one area: ❑ Low back ❑ Leg(s)

Q3. % question ______

**Lumbar Spinal Stenosis (SSHQ)**

❑ Q1 Numbness and/or pain in the thighs down to the calves and shins

❑ Q2 Numbness and/or pain increase in intensity after walking for a while, but are relieved by taking a rest.

❑ Q3 Standing for a while brings on numbness and/or pain in the thighs down to the calves and shins.

❑ Q4 Numbness and/or pain are reduced by bending forward

**5 Repetition Sit-to-Stand Test Time (sec.): _______**

**Low back area:** Pain (pre-test)______ Pain (post-test)______

**Leg area:** Pain (pre-test)______ Pain (post-test)______

**ODI Score: _______%**

**STarT Back Score: Total: __________Subscore:____________**

**Pain Pattern**

❑ Back dominant

❑ Leg dominant 1) unilateral 2) bilateral

❑Leg dominant – nerve root irritation/constant

❑Leg dominant – claudication/intermittent, worse with activity

❑ Symptoms resolving/resolved

**Imaging Findings:**

❑ Central spinal canal stenosis ❑ Foraminal stenosis ❑ Lateral recess stenosis

❑ Disc herniation/bulge ❑ Facet joint cyst

❑ Degenerative Spondylolisthesis ❑ Other_______________________________

**Primary Diagnosis:**

❑1DDD ❑ 2 Mechanical Back Pain ❑ Radiculopathy **3**a. Claudicant type **4**b. Disc-related

❑5 Neurogenic Claudication ❑6 Hip/Knee Osteoarthritis/meralgia ❑7 Other: _____________________

**Triage Outcome:**

**❑ Nonsurgical/does not require in-person consultation (select all that apply):**

- Symptoms resolving/resolved
- Chronic pain not amenable to surgery
- Patient not wanting surgery
- Back dominant pain without treatable structural pathology
- Inadequate conservative treatment
- Mechanical back pain appropriate for referral to physiotherapy
- Symptoms related to a body system or joint, other than spine
- Yellow flags: compensation/litigation issues; pain behaviour
- Other:________________________________________________________

**❑ Appropriate for in-person consultation with APP**

- Back dominant pain (high risk of chronicity/need for investigation to r/o instability)
- Leg dominant pain, (conservative management not maximized)
- Do not want surgery
- Other:________________________________________________________

**❑ Appropriate for Virtual phone call FU**

- Back dominant pain (high risk of chronicity/need for investigation to r/o instability)
- Leg dominant pain, (conservative management not maximized)
- Do not want surgery
- Other:_______________________________________________________

**❑ Appropriate for consultation with surgeon/surgical referral made**

**(select all that apply):**

- Leg-dominant pain, +/- objective neurological loss and concordant imaging
- Structural lesion (spondylolisthesis, lysis/instability)
- Patient considering surgery
- Complexity/atypical findings
- Requiring further investigations
- Red flags
- Other:________________________________________________________
