# Supplementary material for: Efficiency of telephone triage in the assessment of low back pain at a tertiary spine clinic
Source: BMC Musculoskelet Disord. 2025 Dec 17;27:104. doi: 10.1186/s12891-025-09416-y (PMC12874845; doi:10.1186/s12891-025-09416-y)
Supplement: Supplementary file 2 — Supplementary Material 2 [file 12891_2025_9416_MOESM2_ESM.docx]

**Flow Chart**


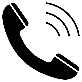


**Results**

**Spine surgery consult**

**n = 2**

**Non-surgical**

**n = 25**

**Non-surgical**

**n = 1**

**Hip arthroplasty surgery**

**n = 3**

**Spine surgery consult**

**n = 1**

**2^nd^ phone call**

**n = 27**

**In-person**

**n = 5**

**Patients**

**n = 100**

**Referred for surgery consult**

**n = 36**

**Triaged as non-surgical**

**n = 64**

**Required follow-up by APP**

- 33 spine surgical consults
- 3 hip arthroplasty surgeries
- 64 non-surgical

{

Total:
